# Nivolumab in gastric cancer with liver metastasis complicated by immune-mediated hepatitis: a case report and FAERS database analysis

**DOI:** 10.3389/fimmu.2025.1663107

**Published:** 2025-10-22

**Authors:** Yong-Li Zhang, Jia-Lan Zhao, Ke-Jun Qu, Zong-Lin Jiao, Yan-Ru Chen, Jia Zhou, Jiang-Lin Li, Jun-Wei Li

**Affiliations:** ^1^ Department of Pharmacy, Shenzhen People’s Hospital (The Second Clinical Medical College, Jinan University, The First Affiliated Hospital, Southern University of Science and Technology), Shenzhen, China; ^2^ Department of Oncology, Shenzhen People’s Hospital (The Second Clinical Medical College, Jinan University, The First Affiliated Hospital, Southern University of Science and Technology), Shenzhen, China; ^3^ Department of Respiratory Medicine, Shenzhen People’s Hospital (The Second Clinical Medical College, Jinan University, The First Affiliated Hospital, Southern University of Science and Technology), Shenzhen, China

**Keywords:** nivolumab, immune checkpoint inhibitor, immune-mediated hepatitis, immunotherapy, adverse events

## Abstract

Nivolumab is a monoclonal antibody that targets the PD-1 pathway, significantly transforming cancer immunotherapy. However, its use is associated with immune-related adverse events (irAEs), including immune-mediated hepatitis (IMH), which can be severe or even life-threatening. We present a case of an 81-year-old male with gastric cancer and liver metastasis, who demonstrated significant anti-tumor efficacy following nivolumab monotherapy. The patient developed grade 3 IMH during treatment, but after discontinuing the medication and receiving timely treatment, his symptoms improved, and liver biochemical markers declined. Additionally, using the FDA Adverse Event Reporting System (FAERS) database, we analyzed the incidence of hepatitis adverse events caused by different immune checkpoint inhibitors (ICIs) in various age groups of patients to better understand the safety of these drugs in different patient populations.

## 1 Introduction

Gastric cancer (GC) remains a significant global health issue, with studies indicating nearly one million new cases and hundreds of thousands of cancer-related deaths annually. It is the third leading cause of cancer-related deaths globally, with high incidence and mortality rates, particularly in East Asia, including China (1). Despite the therapeutic advances in surgery, chemotherapy, and targeted therapies, the prognosis for advanced gastric cancer patients remains poor, with a 5-year overall survival rate of only 5.7%, highlighting the urgent need for new treatment strategies (2).

Immune checkpoint inhibitors (ICIs) are an important therapeutic approach in gastric cancer treatment, enhancing the body’s anti-tumor immune response by blocking immune checkpoint pathways. Monoclonal antibodies targeting Programmed Cell Death 1 (PD-1), such as nivolumab, have become essential drugs in gastric cancer treatment. Nivolumab, a human immunoglobulin G4 (IgG4) monoclonal antibody, specifically blocks the binding of PD-1 to its ligands PD-L1 and PD-L2, relieving the immune suppression of tumor cells on T-cells and restoring the T-cell’s ability to recognize and attack tumor cells, thereby enhancing the anti-tumor immune response (3). However, blocking the key immune regulator PD-1 can lead to excessive immune system activation, resulting in immune-related adverse events (irAEs) (4). Among these, IMH is one of the more severe irAEs, with an incidence rate of 5%-10%, second only to skin toxicity (44%-68%) and gastrointestinal adverse reactions (35%-50%) (5). If not diagnosed or managed properly, IMH can lead to severe liver injury, acute liver failure, or even death, and may result in the discontinuation or failure of immunotherapy (6). Therefore, timely identification and effective management of IMH are critical for improving patient outcomes and quality of life.

This article reports a case of a gastric cancer patient with liver metastasis, who showed good anti-tumor effects after receiving monotherapy with nivolumab. However, during treatment, the patient developed severe IMH. After discontinuing the medication and receiving timely treatment, his symptoms improved, and liver biochemical markers showed a downward trend.

## 2 Case presentation

**Figure 1:**
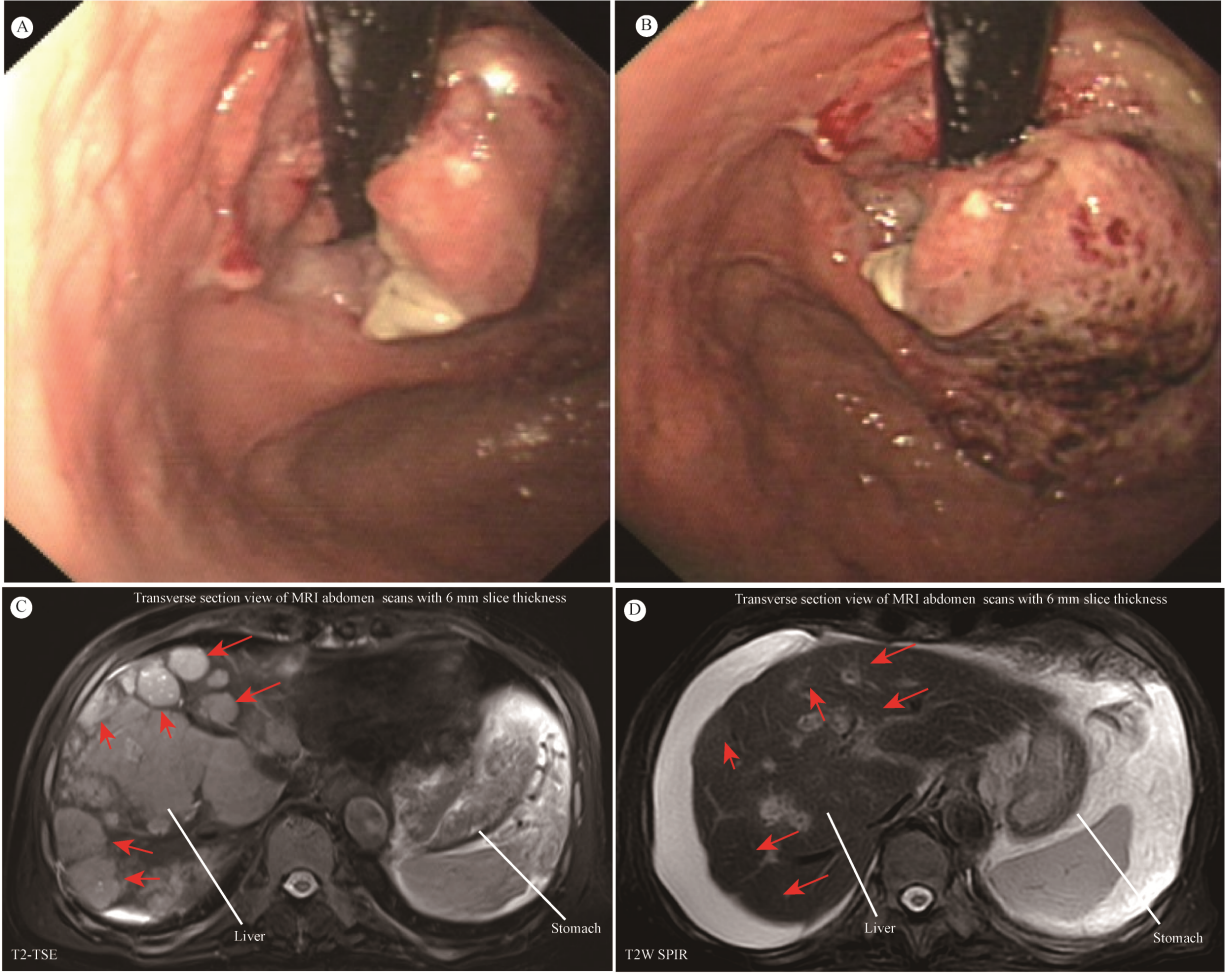
**(A, B)** Giant space-occupying lesion at the gastric fundus. **(C, D)** MRI of the abdomen. Contrast-enhanced MRI of the abdomen showed that the size and number of multiple liver metastatic tumor lesions have notably shrunk after more than four months of monotherapy with nivolumab (red arrow).

The patient, an 81-year-old male, underwent gastroscopy on April 7, 2024, which showed an irregular tumor on the lesser curvature and posterior wall of the gastric fundus. The tumor occupied about one-third of the gastric fundus area, presenting with ulceration, hardening, and active bleeding (**Figures 1A, B**). Enhanced abdominal MRI showed thickening of the gastric wall and multiple hepatic masses (**Figure 1C**). The pathology of the gastric biopsy revealed atypical cell infiltration, indicating a malignant tumor with two components: one part was moderately differentiated adenocarcinoma, while the other component exhibited a solid pattern of poorly differentiated carcinoma with strong neuroendocrine marker expression, suggesting large cell neuroendocrine carcinoma. Immunohistochemistry results were as follows: Adenocarcinoma component: CK8/18(+), CK7(+), Syn(-), EMA(+), Hepatocyte(-), Ki-67(40%+), Her2(1+), MLH1(+), PMS2(+), MSH2(+), MSH6(+), P53(80%+, mutated); Poorly differentiated carcinoma component: CK8/18(+), CK7(-), Syn(strong+), EMA(-), Hepatocyte(-), Ki-67(50%+), Her2(1+), MLH1(+), PMS2(+), MSH2(+), MSH6(+), P53(80%+, mutated); PD-L1(BP6099, TC score: 5%+). The diagnosis was stage IV gastric cancer with multiple hepatic metastases. Considering the patient’s advanced age, history of diabetes, coronary artery disease, chronic kidney disease, and poor liver and kidney function, nivolumab (240 mg q2w) monotherapy was initiated on April 9, 2024. After more than four months of monotherapy with nivolumab, contrast-enhanced MRI imaging demonstrates significant interval regression of multiple hepatic metastases and a substantial reduction in portal vein thrombus burden compared with prior examinations. Improved visualization of the middle and left hepatic veins is noted. The gastric wall exhibits mild thickening, which represents a mild improvement relative to previous findings. Furthermore, the mass at the gastric fundus demonstrates an interval reduction in size (**Figure 1D**).

**Figure 2:**
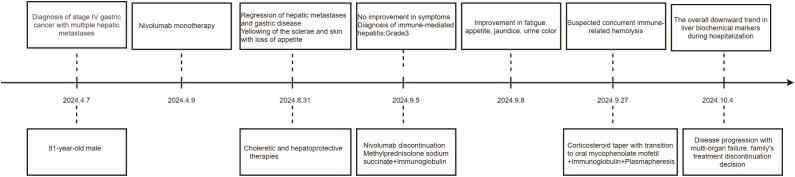
Timeline of each treatment.

However, on August 31, 2024, the patient developed scleral and generalized skin jaundice, as well as a decreased appetite. Liver function tests indicated: total bilirubin (T-BIL) 132.9 *μ*mol/L↑, alanine aminotransferase (ALT) 232 U/L↑, aspartate aminotransferase (AST) 316 U/L↑, alkaline phosphatase (ALP) 666 U/L↑, γ-glutamyl transpeptidase (GGT) 480 U/L↑. MRCP showed cirrhosis, ascites, splenomegaly, and varices at the porta hepatis and gastric fundus. The patient received choleretic and hepatoprotective therapies. However, no improvement in symptoms was observed. On September 5, 2024, the patient was diagnosed with immune checkpoint inhibitor-associated hepatitis, with a severity level of 3. Nivolumab was immediately discontinued, and methylprednisolone sodium succinate (120mg, iv, qd) was administered and gradually reduced over 4 weeks. Concurrently, intravenous immunoglobulin (10mg, iv, qd) was given. Liver protection, nutritional support, and symptomatic treatment were also provided. After two days of treatment with glucocorticoids and intravenous immunoglobulin, the patient’s fatigue and decreased appetite improved, and the scleral and skin jaundice as well as urine color lightened. Liver function tests also showed improvement. On September 27, 2024, considering the possibility of immune-related hemolysis, the methylprednisolone sodium succinate dose was gradually tapered off, and the patient was switched to oral mycophenolate mofetil (0.5-0.75mg, po, bid), continuing intravenous immunoglobulin therapy, and undergoing plasmapheresis. During the hospitalization, the liver biochemical markers showed an overall downward trend (as shown in **Table 1**). The patient was later transferred to the ICU for continued treatment. On October 4, 2024, due to disease progression and multi-organ failure, the family ultimately decided to discontinue treatment and the patient was discharged. The timeline is shown in **Figure 2**.

**Table 1:** Results of liver function analysis during hospitalization.

Date	T-BIL (*μ*mol/L)	ALT (U/L)	AST (U/L)	ALP (U/L)	GGT (U/L)
31 August	132.9	232	316	666	480
6 September	237.1	163	207	711	444
10 September	155.4	370	390	548	482
13 September	161.2	400	301	443	512
17 September	135.4	98	58	97	365
23 September	99.9	189	133	229	356
27 September	121.4	155	92	307	374
28 September	168.9	108	87.3	267	339
29 September	114.6	88.8	61.5	230	297
30 September	83.1	57.8	46.3	170	193
3 October	89.8	18.8	38.4	153	213

T-BIL, total bilirubin; ALT, alanine aminotransferase; AST, aspartate aminotransferase; ALP, alkaline phosphatase; GGT, γ-glutamyl transpeptidase.

## 3 FAERS database analysis of the relationship between different ICIs and risk of IMH in age groups

Based on the FAERS data collected from OpenVigil 2.1-MedDRA-v24 (data 2004Q1-2024Q2), we analyzed the hepatitis adverse events caused by different immune checkpoint inhibitors (ICIs) in patients of different age groups (see **Tables 2**, **3**). The data show that nivolumab and pembrolizumab have the highest number of hepatitis reports across all age groups, with 467 and 504 cases respectively. Moreover, in specific age groups, the statistical indicators such as proportional reporting ratio (PRR) and reporting odds ratio (ROR) are also relatively high (for nivolumab in the age group of 60 to 110 years old, PRR: 7.103, ROR: 7.155; for pembrolizumab in the age group of 30 to 60 years old, PRR: 7.332, ROR: 7.407). This indicates that the use of nivolumab and pembrolizumab is associated with an increased risk of hepatitis. In addition, although the number of reports for durvalumab, avelumab, and atezolizumab is relatively low, the PRR and ROR values in certain age groups also show potential associations. However, due to the small sample size, the stability and reliability of these results may need further data to support. Overall, these data suggest that we need to closely monitor hepatitis adverse events in patients of all age groups when using ICIs.

**Table 2:** The incidence numbers and rates of hepatitis across different age groups associated with ICIs.

Adverse	Drug	0–30 years	30–60 years	60–110 years	All ages
		N (%)	N (%)	N (%)	N (%)
Hepatitis	nivolumab	9 (1.167)	110 (1.111)	189 (0.854)	467 (1.005)
	pembrolizumab	3 (0.637)	128 (1.166)	194 (0.814)	504 (1.067)
	durvalumab	0 (0)	5 (0.469)	11 (0.316)	61 (0.891)
	avelumab	0 (0)	2 (0.76)	4 (0.365)	32 (1.713)
	atezolizumab	0 (0)	24 (0.677)	55 (0.568)	212 (1.189)
	cemiplimab	0 (0)	0 (0)	2 (0.593)	15 (1.296)
	dostarlimab	0 (0)	0 (0)	1 (1.408)	3 (0.673)
	tislelizumab	0 (0)	0 (0)	1(0.524)	7 (0.688)
	retifanlimab	0 (0)	0 (0)	1 (25.0)	1 (3.704)
	serplulimab	0 (0)	0 (0)	1 (11.111)	2 (16.667)
	toripalimab	0 (0)	0 (0)	0 (0)	3 (0.706)

**Table 3:** The association between hepatitis occurrence and ICIs in various age groups.

Drug	0-30 years		30-60 years		60-110 years		All ages	
	PRR (χ^2^)	ROR	PRR (χ^2^)	ROR	PRR (χ^2^)	ROR	PRR(χ^2^)	ROR
nivolumab	6.77 (38.525)	6.838	6.959 (542.974)	7.026	7.103 (938.144)	7.155	9.583 (3462.77)	9.67
Pembrolizumab	3.682 (3.483)	3.699	7.332 (675.445)	7.407	6.779 (903.587)	6.826	10.208 (4027.299)	10.307
durvalumab	0 (9.348)	0	2.877 (4.393)	2.886	2.526 (8.658)	2.531	8.274 (381.528)	8.339
avelumab	0 (23.101)	0	4.668 (2.683)	4.696	2.913 (3.294)	2.92	15.874 (430.702)	16.134
atezolizumab	0 (0.387)	0	4.172 (54.545)	4.193	4.58 (148.414)	4.601	11.155(1921.466)	11.277
cemiplimab	0 (35.144)	0	0 (0.786)	0	4.731 (2.747)	4.754	12.0 (140.441)	12.144
dostarlimab	0 (143.556)	0	0 (6.713)	0	11.226 (1.897)	11.373	6.221 (8.449)	6.256
tislelizumab	0 (27.918)	0	0 (0.216)	0	4.173 (0.283)	4.19	6.361 (26.507)	6.398
retifanlimab	0 (71.28)	0	0 (8.627)	0	199.274 (48.872)	265.365	34.248 (7.599)	35.527
serplulimab	0 (0)	0	0 (152.646)	0	88.566 (21.174)	99.512	154.129 (170.567)	184.755
toripalimab	0 (47.189)	0	0 (0.802)	0	0 (1.976)	0	6.528 (9.067)	6.567

Using the FDA Adverse Event Reporting System (FAERS) data from 2004Q1-2024Q2, ICIs, Immune Checkpoint Inhibitors; PRR, proportional reporting ratio; ROR, reporting odds ratio; Q1, first quarter.

## 4 Discussion

This case involves an 81-year-old male patient with advanced gastric cancer and liver metastasis, exhibiting microsatellite stability and positive PD-L1 expression (TC score 5%+). Given the patient’s advanced age, multiple comorbidities, impaired liver and kidney function, and a TC score≥1%, single-agent nivolumab was chosen for treatment. After four months of treatment, the gastric body tumor reduced in size, and the multiple liver metastases showed significant shrinkage and decrease, yielding favorable therapeutic results. This case report indicates that nivolumab monotherapy can serve as an effective treatment for elderly patients with gastric cancer and multiple liver metastases.

The ATTRACTION-2 trial demonstrated the efficacy of nivolumab monotherapy in patients with advanced gastric cancer (GC). This trial reported a median overall survival (OS) of 5.3 months, a median progression-free survival (PFS) of 1.6 months, and a response rate (RR) of 11.2% (7). In 2-year update date, the median OS of responding patients with a complete response (CR) or partial response (PR) was 26.6 months, with a 12-month survival rate was 87.1%, and a 24-month survival rate was 61.3% (8). The observational study of nivolumab monotherapy, the DELIVER trial, showed OS, PFS, and RR similar to the results of the ATTRACTION-2 trial, presenting that nivolumab monotherapy would be as effective in clinical practice as in clinical trials. In addition, the exploratory analysis indicated that increasing depth of response was associated with longer median PFS and OS in nivolumab treatment at a later-line setting (9). The CheckMate 649 trial demonstrated that nivolumab plus chemotherapy significantly extended both OS and PFS compared with chemotherapy in patients with advanced GC, particularly among those with PD-L1 combined positive score (CPS)≥5 (10). A meta-analysis of randomized controlled trials showed that both nivolumab monotherapy and nivolumab plus chemotherapy significantly improved OS and PFS in patients with advanced GC, highlighting their potential to enhance prognosis in advanced disease (11). Furthermore, nivolumab monotherapy may be more suitable for patients with advanced age, poor performance status, or multiple comorbidities who may not tolerate the toxicities associated with combination therapy (11).

Although the liver is a common metastatic site of GC, the treatment for liver metastasis of GC has not been well established. The current guidelines for GC recommend nivolumab combined with chemotherapy as first-line regimen and nivolumab monotherapy as third-line regimen for advanced or metastatic GC with HER2-negative, including liver metastasis (12, 13). Despite the limited efficacy of nivolumab monotherapy in GC treatment, some case reports indicated that the primary tumor dramatically shrank and liver metastases shrank or disappeared in GC patients with multiple liver metastases receiving nivolumab as third-line regimen. Moreover, in these case reports, patients achieved PR or CR with nivolumab treatment (14–18).

Immunotherapy has provided significant survival benefits for patients with advanced gastric cancer, but the associated hepatotoxicity cannot be overlooked. Although the activation and expansion of T cells through the lack of negative regulation by PD-1 and CTLA-4 are considered the immune pathogenesis of irAEs, the molecular mechanisms underlying the development of liver irAE remain unclear (19). The incidence of liver irAEs caused by nivolumab monotherapy is reported to be 6.4%, and liver damage induced by nivolumab typically occurs 8–12 weeks after the first injection (20, 21).

IMH is a special type of drug-induced liver injury, commonly presenting as asymptomatic elevation of AST and ALT, with or without bilirubin elevation. It may also present with nonspecific symptoms such as fever, fatigue, nausea, vomiting, and loss of appetite. When bilirubin increases, jaundice of the skin and sclera, and tea-colored urine may be observed (22). The diagnosis of IMH is a diagnosis of exclusion. Firstly, the patient has not used any known drugs that are clearly associated with liver damage prior to treatment. Secondly, liver injury caused by other factors such as secondary liver tumor progression, acute viral hepatitis, and autoimmune hepatitis is excluded. Additionally, symptoms improve and liver biochemical markers trend downwards after discontinuation of the drug and timely treatment. This case meets the above diagnostic criteria, and thus, the patient was diagnosed with nivolumab-induced immune-related hepatitis. Notably, our analysis based on the FDA Adverse Event Reporting System (FAERS) database reveals that, compared to other age groups, nivolumab-induced immune-related hepatitis is most frequently reported in individuals aged 60 and above, with the strongest association to this adverse reaction. This finding suggests that advanced age may be a risk factor, and thus, clinicians should remain vigilant and enhance drug monitoring in this population.

The management strategy for nivolumab-induced hepatotoxicity should be graded according to the severity of liver toxicity. Corticosteroids are the first-line treatment for irAEs, with rapid and potent anti-inflammatory effects, and can treat the majority of IMH cases (22–24). For patients with grade 3 or 4 IMH, if there is no significant improvement after 1–2 days of corticosteroid treatment, consideration should be given to adding mycophenolate mofetil or other immunosuppressive therapies, including antithymocyte globulin, azathioprine, tacrolimus, or tocilizumab (23, 24). Additionally, in clinical practice, early intravenous administration of intravenous immunoglobulin (IVIG) for IMH, particularly in patients with grade 3 or higher, has been shown to help improve prognosis (22).

## 5 Conclusion

We present a case of gastric cancer with liver metastasis, treated with nivolumab alone, which led to a reduction in both the primary and liver metastatic lesions, demonstrating the therapeutic potential of this approach for such patients. However, during treatment, the patient developed severe IMH, which showed improvement after prompt drug cessation and aggressive management. Consequently, clinicians should exercise high caution and carefully monitor liver function during ICI treatment, enabling early diagnosis and prompt treatment. Particularly in older patients, there should be enhanced monitoring of drug use and regular evaluations.

## Data Availability

The original contributions presented in the study are included in the article/Supplementary Material. Further inquiries can be directed to the corresponding author.
